# Acceptability and Preferences among Men and Women for Male Involvement in Antenatal Care

**DOI:** 10.1155/2017/4758017

**Published:** 2017-01-24

**Authors:** Nompumelelo Yende, Annelies Van Rie, Nora S. West, Jean Bassett, Sheree R. Schwartz

**Affiliations:** ^1^Witkoppen Health and Welfare Centre, Johannesburg, South Africa; ^2^Department of Epidemiology, University of North Carolina Gillings School of Global Health, Chapel Hill, NC, USA; ^3^Department of Epidemiology, Johns Hopkins Bloomberg School of Public Health, Baltimore, MD, USA

## Abstract

*Introduction*. Male involvement in antenatal care (ANC) has been associated with improved prevention of mother-to-child transmission outcomes in Sub-Saharan Africa; yet it remains uncommon. We assess acceptability of male involvement from the male and female perspectives and potential incentives for men to attend ANC.* Methods*. Adult pregnant women and men attending primary healthcare at Witkoppen Health and Welfare Centre in Johannesburg, South Africa, from October 2013 to January 2014, were recruited using stratified random sampling to ensure equal representation across gender and HIV status.* Results*. 300/332 individuals (93.8%) offered participation consented. Among the 150 women, 97% had a partner; the majority (92%) preferred partner attendance at ANC, and 14% reported partner attendance during this pregnancy. The 150 men had low knowledge of services rendered at ANC outside of pregnancy monitoring, and few (19%) had previously attended ANC. Blood pressure screening, fatherhood information, and HIV testing were identified by men as incentives for attendance. Women and men expressed high willingness to, respectively, deliver (95%) and respond (97%) to ANC letter invitations.* Conclusion*. Invitation letters to promote male involvement in ANC are highly acceptable to pregnant women and men. Focusing invitation messages on fatherhood and primary healthcare rather than HIV testing may provide greater motivation for male involvement.

## 1. Introduction

Improved engagement in antenatal care (ANC) and prevention of mother-to-child transmission (PMTCT) programmes during pregnancy have resulted in substantial reductions in mother-to-child transmission of HIV in South Africa, but loss to follow-up from antiretroviral therapy (ART) programmes is prominent, especially in the postpartum period and among women newly diagnosed with HIV during pregnancy [1, 2]. One contributing factor may be that women who have not disclosed their HIV status to a partner or family members can no longer attribute to pregnancy regular clinic visits and daily medication intake during the postpartum period [3]. Interventions to retain women in ART care during pregnancy and postpartum are thus needed to ensure optimal health outcomes for the mother and child [4, 5].

Because men have social and economic roles within families that can influence decisions related to the health of the mother and child, participation of men in ANC and family health is one potential solution to improve PMTCT outcomes [6–8]. A study in Kenya found that male involvement was associated with increased adherence to maternal ART prophylaxis, better adherence to infant feeding methods, and a 45% decrease in risk of HIV transmission from mothers to infants over a one-year period [6]. Testing couples in ANC may provide a mechanism through which HIV-infected women can disclose and/or receive treatment support from their partners. Similarly, when the female partner tests negative, couples testing can help to identify risks for acute infection during pregnancy.

Several barriers result in poor ANC attendance by male partners [9, 10]. Throughout much of the world maternal and infant health are perceived as women's responsibilities and men often do not feel welcome at ANC as no other men are in ANC waiting rooms, negative healthcare provider treatment of male partners, and long wait times [9, 11–14]. In addition, men may have employment responsibilities resulting in opportunity-costs of lost wages.

Invitation letters have been suggested as one potential approach to increase male involvement [10], but few data are available on the acceptability to men and women of such letters and their effect on HIV testing or retention in care [15]. To inform the design of a male involvement intervention, we assessed the acceptability of male involvement in PMTCT and the willingness of women and men to distribute and respond to a letter invitation, as well as preferences for specific elements of a male involvement invitation letter.

## 2. Methods

### 2.1. Study Design and Population

Participants were enrolled in a cross-sectional study at Witkoppen Health and Welfare Centre (Witkoppen), a primary health clinic in Johannesburg, South Africa, between October 2013 and January 2014. Every third man aged 18–59 years receiving care at the HIV clinic and every fifth man receiving general non-HIV care for issues other than acute illnesses were screened for eligibility and invited to participate if eligible. Every third pregnant woman aged ≥18 years known to be HIV positive at enrolment and every fifth HIV negative pregnant woman were eligible to participate if they were attending the antenatal clinic. A stratified sampling strategy was used to ensure that an equal number of men and women (*n* = 150 each), as well as equal numbers of people living with and without HIV (*n* = 150 each), were included.

Trained research assistants administered a one-time, anonymous, structured questionnaire in English, isiZulu, or SeSotho to collect data on demographics, reproductive and HIV-related history, and knowledge, acceptability, and preferences related to male participation in ANC. Participants were also asked to review two invitation letters and indicate which letter they preferred and to assess specific passages of the letters (see Appendix for Supplementary Materials available online at https://doi.org/10.1155/2017/4758017). Passages related to reasons for attending ANC and activities that would be undertaken at ANC. Reading comprehension was assessed for all participants and letters were read aloud to participants in cases of poor comprehension.

### 2.2. Ethics, Consents, and Permissions

This study was approved by the Human Research Ethics Committee at the University of the Witwatersrand in Johannesburg, South Africa, and the Institutional Review Board at the University of North Carolina at Chapel Hill in the United States. All participants provided written informed consent.

### 2.3. Statistical Analyses

Differences in the distribution of characteristics between groups were compared using Fisher's exact tests and Wilcoxon rank sum tests. The study had 80% power to detect a >1.5-fold difference in acceptability of male involvement between men and women, assuming at least 40% of all participants found male involvement to be acceptable. All analyses were conducted in Stata 12.1* (College Station, TX, USA)*.

## 3. Results

Of the 332 individuals invited, 300 (90.4%) agreed to participate. Reasons for attending Witkoppen clinic among the 150 men enrolled were not feeling well (*n* = 58, 38.7%), HIV treatment (*n* = 53, 35.3%), routine checkup (*n* = 28, 18.7%), HIV testing (*n* = 7, 4.7%), or other (*n* = 4, 2.6%). Among the 150 pregnant women receiving ANC, the median gestational age was 24 weeks (interquartile range [IQR] 17–31).

Participating men were older, were more likely to be employed, had higher incomes, had attained a lower education level, and had more children than participating women (Table 1). By design, 50% of all men and women were HIV positive; more men than women were unaware of their HIV status (16.7% versus 6.0%, *p* < 0.01). Women were more likely than men to be in a relationship (97.3% versus 87.3%, *p* < 0.01) with an average 6-year duration of the relationship (5 years for women, 7 years for men, *p* < 0.01). The majority of participants (81.6%) had disclosed their status to their partner, with similar proportion of men and women having disclosed to their partners.

### 3.1. Experience with Male Involvement

Few (*n* = 21/146, 14%) women with a current partner reported that their male partner had attended an ANC visit with them during the current pregnancy, and 20% (*n* = 29/147) of men reported prior ANC attendance with a partner (Table 1). Characteristics of men who had and had not attended ANC were similar except for a higher level of education among men who had ever attended an ANC visit (Table 2). Among HIV positive men, 100% of those who had attended ANC had disclosed their HIV status to their recent partner compared to 82% disclosure rate among those who have never attended an ANC visit, though the difference was not statistically significant (*p* = 0.19).

Even though two-thirds of women (*n* = 96/143, 65.0%, data missing for 3 women with partners) had invited their male partner to accompany them to an ANC visit, only 21 (14.4%) women reported that their male partner had actually accompanied them during their current pregnancy. Women who had or had not invited a partner to attend ANC were similar, with the exception that fewer women who received their HIV diagnosis during pregnancy had invited a male partner to an ANC visit (45.8% versus 14.3%, *p* < 0.01, Table 2).

### 3.2. Knowledge, Acceptability, and Preferences for Male Involvement in ANC

**Figure 1:**
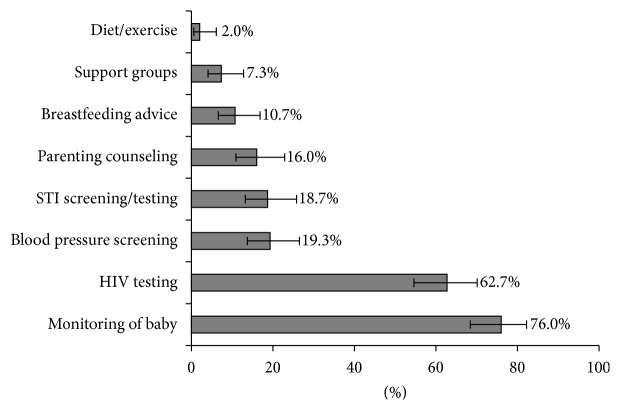
Men's knowledge of services provided to women at antenatal care.

**Figure 2:**
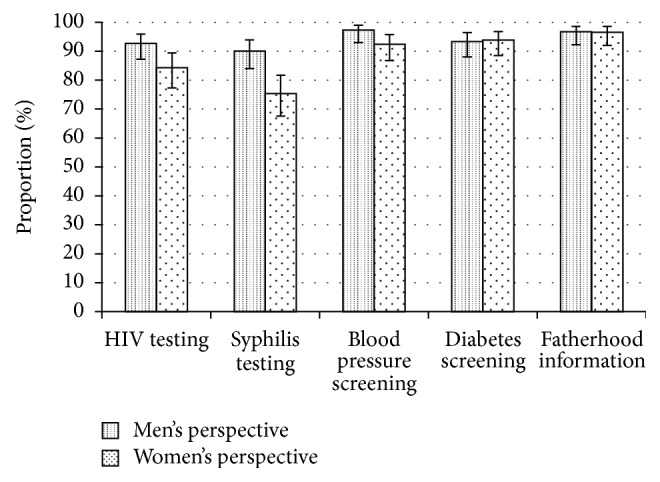
Incentives for male attendance at antenatal care. Men's and women's perspectives of what healthcare services could be provided to men at the antenatal clinic as an incentive to come to the clinic with their partners.

The majority of men knew that monitoring the baby's progress (76%) and maternal HIV testing (63%) are part of ANC services (Figure 1). Other ANC activities, such as breastfeeding and nutrition counselling, STI screening, and blood pressure screening, were known by less than 20% of men. Both men and women believed that services for fathers as part of ANC could encourage them to attend. Almost all men (≥90%) were interested in fatherhood information, HIV testing, and screening for blood pressure, syphilis, and diabetes (Figure 2). Women were less likely than men to report HIV and syphilis testing as incentives for male involvement in ANC (Figure 2, *p* = 0.03 and *p* < 0.01, resp.).

Almost all (92%, *n* = 133/145 women in relationships, missing for *n* = 1) women reported they would like their partners to attend their ANC visit and 95% (138/145) would give their partners letter invitations. Among HIV negative women and HIV positive women who had not yet disclosed their status to their partner (*n* = 87), 95% preferred couples testing in ANC to individual HIV testing. Most men (95%, *n* = 142/149, missing for *n* = 1) believed that their partners would want them to attend ANC if given the option and almost all (97%, 145/150) men said they would attend an ANC visit if they received a letter invitation from their partners. Among the few (2.7%, 8/150) individuals not willing to participate in an ANC invitation programme, almost all (7/8) were women, of which 3 were HIV positive and 4 were HIV negative.

Men identified time off from work as their greatest barrier to attending ANC with a partner (38%), followed by no other men at the clinic (14%), transport cost (13%), and long wait times (10%). Less than five percent of men listed cultural/religious beliefs, partners' preferences, or clinic hours as the main barrier to attending ANC. Twenty percent of men reported no barrier to attending ANC. Men preferred Saturdays and early weekday mornings as optimal attendance hours. Most men (96%) were in favour of a “Father's Day” at the clinic in which men would attend ANC on the same day and multiple activities for fathers-to-be would be offered on those days.

Men and women read over sample ANC male invitation letters (see online supplement). While 70% of women expressed a preference for one of the two letters, men were almost split equally (46 versus 54%) in their preference for the letters. The majority of men (55%) preferred positive messages related to fatherhood and being a supportive partner rather than an emphasis on prior research findings suggesting improved maternal and child health outcomes from male involvement; most women (65%) preferred the latter (Table 3). Both men (53%) and women (68%) preferred a more general description of counselling and health assessments offered to men at ANC rather than a focus on testing for HIV and sexually transmitted infections. No differences were observed by HIV status of the participant* (results not shown)*.

## 4. Discussion

We found a high willingness among both women and men, independent of HIV status, to embrace male involvement in ANC. These findings echo those from a study in Cameroon indicating high acceptability among women for male involvement in ANC [16]. Despite high acceptability, only 14% of women reported that a partner had attended ANC with them during their current pregnancy. This baseline level of male partner attendance is in line with numbers reported in a study from Malawi, though lower than what has been observed in several other Sub-Saharan African studies [17, 18]. Both men and women expressed high interest in the use of invitation letters for male involvement and perceived information about fatherhood, general health screening, and HIV and STI screening as incentives for male involvement in ANC.

Like others, we found that work and financial considerations were barriers to male ANC attendance [9]. Weekend clinic hours, earlier morning hours, shorter clinic wait times, fast-tracking men attending ANC with their partners, and/or “Father's Days” could provide solutions [19, 20]. Care must however be taken that encouragement of male partner attendance does not result in healthcare providers rejecting or stigmatizing women who do not attend with partners as documented in Tanzania [21]. We also observed that men had little knowledge of what occurs at ANC visits, which may result in disinterest among men and has been reported in other studies as well [12, 19, 22]. Our data support the suggestion made by Koo et al. that inviting male partners and highlighting their importance at ANC may help to shift attitudes and generate a more welcoming environment for male partners [23]. Evidence from Malawi also suggests that structural changes to make ANC more male-friendly can result in greater male participation [17].

Our findings further suggest that men and women perceive health assessments for men as incentives for male ANC involvement. In our study, women were less inclined than men to emphasize HIV testing in the invitation letter. This may be because women were less comfortable with giving their partner a letter that invites them for HIV testing, or it may be that women perceive their partners as less likely to attend if the invitation letter specifically highlights HIV testing. A randomized control trial involving a letter invitation in Tanzania found decreased HIV testing among women attending ANC with their partners as compared to women randomized to receive individual testing, reinforcing that women's comfort with any male involvement intervention is essential [24].

Our study was specifically designed to access acceptability and preferences around male involvement. A study strength is that both men and women were included, with representation of both HIV positive and HIV negative participants. Inclusion of both sexes is critical as even if men are amenable to attending, male involvement is unlikely to occur if the woman does not want or invite him to participate in care. There were however some limitations to this analysis. While men were included, we did not specifically recruit men with currently pregnant partners and thus opinions of the participating men will not precisely reflect those of men whose partners are currently pregnant. We could not verify the accuracy of reports by men and women regarding prior male involvement. Furthermore, given that women were at varying gestational ages during enrolment, the number of women with male partner attendance during the current pregnancy may be underestimated as she was not yet at her final ANC visit. Results from the study are from a single clinic and thus may not be generalizable to the population more broadly. Finally, enrolling men at a clinic may result in a biased response, as these men may be more likely to engage in positive health seeking behaviour.

## 5. Conclusions

Increasing male involvement in PMTCT programming has been shown to improve health outcomes for the mother and child [6, 25]. Male participation in ANC is highly acceptable to both men and women but rarely practiced. Interventions should make ANC male-friendly, emphasize development of parenting skills, raise the perception that ANC is not a woman's activity but is about promoting family health, and provide comprehensive incentives for male partner attendance, including integrated health services for men. This may motivate women to invite men, as well as encourage men's attendance. Future studies should assess the impact of such interventions on male involvement as well as maternal retention and treatment adherence within PMTCT to determine their true value.

## Supplementary Material

The supplementary material provides the two sample letters that participants were shown and read. Participants chose which overall letter and which paragraphs they preferred from each letter in order to assess patient preferences for messaging around male involvement in antenatal care.

## Figures and Tables

**Table 1:** Characteristics of men and women interviewed about acceptability of male involvement in antenatal care.

Characteristics	Overall (*n* = 300)	Men (*n* = 150)	Women (*n* = 150)	*p* value
Age in years, *n* (%)				
18–24	52 (17.3)	9 (6.0)	43 (28.7)	<0.01
25–34	132 (44.0)	49 (32.7)	83 (55.3)	
35 and over	116 (38.7)	92 (61.3)	24 (16.0)	

Employment, *n* (%)	166 (55.3)	107 (71.3)	59 (39.3)	<0.01

Median monthly income, USD [IQR]^*∗*^	294 [245–392]	343 [245–441]	275 [216–343]	<0.01

Education, *n* (%)				
None	4 (1.3)	4 (2.7)	0 (0.0)	<0.01
Primary school	72 (24.0)	52 (34.7)	20 (13.3)	
Secondary school	132 (44.0)	63 (42.0)	69 (46.0)	
Matriculated secondary/tertiary	02 (30.7)	31 (20.6)	61 (40.7)	

Country of origin, *n* (%)				
South Africa	171 (57.0)	81 (54.0)	90 (60.0)	0.10
Zimbabwe	86 (28.7)	41 (27.3)	45 (30.0)	
Other	43 (14.3)	28 (18.7)	15 (10.0)	

Number of living children, *n* (%)				
None	53 (17.7)	15 (10.0)	38 (25.3)	<0.01
One	98 (32.7)	36 (24.0)	62 (41.3)	
Two or more	149 (49.6)	99 (66.0)	50 (33.4)	

HIV status, *n* (%)				
HIV positive	150 (50.0)	75 (50.0)	75 (50.0)	<0.01
HIV negative	16 (38.7)	50 (33.3)	66 (44.0)	
Unknown	34 (11.3)	25 (16.7)	9 (6.0)	

Currently in a relationship, *n* (%)	277 (92.3)	131 (87.3)	146 (97.3)	<0.01

Median age of partner, years [IQR]^†, ‡^	32 [28–37]	32 [26–37]	32 [28–36]	0.84

Mean years in relationship with main partner, [IQR]^†^	6 [3–11]	7 [5–15]	5 [2–8]	<0.01

Male partner attended antenatal care during current pregnancy, *n* (%)	—	—	21 (14.4)	—

Attended antenatal care with a female partner in the past, *n* (%)^§^	—	29 (19.7)	—	—

Disclosed HIV status to most recent partner, *n* (%)^§,¶^	120 (81.6)	62 (84.9)	58 (78.4)	0.40

Want male partners to attend/perceive that female partners would want them to attend ^||,*∗∗*^	275 (93.5%)	142 (95.3%)	133 (91.7)	0.24

Willing to distribute/respond to ANC invitation letter, *n* (%)^††^				
Yes	283 (95.9)	145 (96.6)	138 (95.2)	0.01
No	8 (2.7)	1 (0.7)	7 (4.8)	
Not sure	4 (1.4)	4 (2.7)	0 (0.0)	

*p* values comparing distributions and medians were estimated using Fisher's exact tests and Wilcoxon rank-sum tests, respectively. ^*∗*^Among those employed (*n* = 166); ^†^among those in a relationship (*n* = 277); ^‡^missing for *n* = 1; ^§^missing for *n* = 3; ^¶^among those living with HIV; ^||^among women in relationships; ^*∗∗*^missing for *n* = 1 man and *n* = 1 woman; ^††^missing for *n* = 5.

**Table 2:** Characteristics of men's attendance and women's invitation of men to antenatal care (*n* = 294).

	Men (*n* = 147)			Women (*n* = 147)		
Characteristics	History of attending ANC (*n* = 29)	Never attended ANC (*n* = 118)	*p* value	Women who invited partner to ANC (*n* = 96)	Women who had not invited partner to ANC (*n* = 51)	*p* value
Age in years, *n* (%)						
18–24	3 (10.3)	6 (5.1)	0.45	23 (24.0)	19 (37.3)	0.16
25–34	10 (34.5)	36 (30.5)		58 (60.4)	23 (45.1)	
35 and over	16 (55.2)	76 (64.4)		15 (15.6)	9 (17.6)	

Education, *n* (%)						
None/primary	12 (41.4)	43 (36.4)	0.03	16 (16.7)	4 (7.8)	0.10
Secondary	7 (24.1)	56 (47.5)		38 (39.6)	29 (56.9)	
Matriculated secondary/tertiary	10 (34.5)	19 (16.1)		42 (43.7)	18 (35.3)	

Country of origin, *n* (%)						
South Africa	16 (55.2)	63 (53.4)	0.52	56 (58.3)	32 (62.7)	0.50
Zimbabwe	6 (20.7)	35 (29.7)		28 (29.2)	16 (31.4)	
Other	7 (24.1)	20 (16.9)		12 (12.5)	3 (5.9)	

Number of living children, *n* (%)^*∗*^						
None	—	—	0.48	24 (25.0)	13 (25.5)	0.82
One	6 (20.7)	29 (28.2)		39 (40.6)	23 (45.1)	
Two or more	23 (79.3)	74 (71.8)		33 (34.4)	15 (29.4)	

Talk to partner about HIV, *n* (%)^†,‡^						
Yes	26 (92.0)	89 (89.0)	0.73	87 (93.5)	38 (84.4)	0.12
No	2 (7.1)	11 (11.0)		6 (6.5)	7 (15.6)	

HIV status, *n* (%)						
HIV positive	12 (41.4)	63 (53.4)	0.30	50 (52.1)	24 (47.1)	0.61
HIV negative/unknown	17 (58.6)	55 (46.6)		46 (47.9)	27 (52.9)	

Disclosed HIV status to most recent partner, *n* (%)^§,¶^						
Yes	12 (100.0)	50 (82.0)	0.19	41 (83.7)	16 (66.7)	0.13
No	0 (0.0)	11 (18.0)		8 (16.3)	8 (33.3)	

Relationship duration with partner, *n* (%)^†^						
<2 years	1 (3.6)	8 (8.0)	0.68	15 (15.6)	10 (21.3)	0.48
≥2 years	27 (96.4)	92 (92.0)		81 (84.4)	37 (78.7)	

Pregnant at time of HIV diagnosis^||,*∗∗*^	—	—	—			
Yes	—	—	—	—	13 (54.2)	<0.01
No	—	—	—	—	11 (45.8)	

Level of financial support from partner^†,*∗∗*^	—	—	—			
None/some	—	—	—	34 (36.2)	21 (44.7)	0.36
A lot of support	—	—	—	60 (63.8)	26 (55.3)	

Level of emotional support from partner^†, ††^	—	—	—			
None/some	—	—	—	42 (45.2)	23 (48.9)	0.72
A lot of support	—	—	—	51 (54.8)	24 (51.1)	

*p* values were estimated using Fisher's exact tests. Invitation data were missing for 3 men (*n* = 147) and 3 women (*n* = 147). ^*∗*^Among men with living children (*n* = 132). ^†^Among those in relationships: men, *n* = 128; women, *n* = 143 in relationships; ^‡^missing for *n* = 5/143 women in relationships; ^§^among those in relationships and known to be living with HIV: men, *n* = 73; women, *n* = 74; ^¶^missing for *n* = 1 woman; ^||^among women living with HIV, *n* = 75; ^*∗∗*^missing for *n* = 2; ^††^missing for *n* = 3.

**Table 3:** Preferences among men and women for different components of male ANC invitation letters.

Letter parts	Text from Letter “A”	Text from Letter “B”	Male preferences	Female preferences	*p* value assessing difference in preferences
*Why to come to ANC?*	*Why should you come for the visit? *Your child's health also depends on you, even before your child has been born. A good father supports his partner in attending ANC and getting tested along with her.	*Why should you come for the visit? *Research shows that when fathers attend antenatal care with their partners, both mothers and babies have better health outcomes. Your child's health also depends on you, even before your child has been born.	55% of men prefer wording in * Letter A*	65% of women prefer wording in *Letter B*	<0.01

*What will happen at ANC?*	*What will happen during the visit? *We will provide special counselling for fathers who are expecting a baby so that you know how to support your partner and your new baby. You will be offered testing for HIV and also other Sexually Transmitted Infections (STI) You will receive counselling on how to keep yourself and your child healthy and prevent your child from getting HIV.	*What will happen during the visit? *You will accompany your partner and learn about your baby. We will provide special counselling for fathers who are expecting a baby so that you know how to support your partner and your new baby. Your health affects your ability to be a good parent. Thus, along with making sure that your partner and child are healthy, we will offer you a basic health assessment, including blood pressure reading and screening for infections that can be treated.	53% of men prefer wording in * Letter B*	68% of women prefer wording in *Letter B*	0.01

*Overall letter*	See Appendix A for sample letters. Note that overall 92.0% of men and 98.7% of women were able to read the letter.		54% of men prefer *Letter B* overall	70% of women prefer * Letter B* overall	<0.01
