# Supplementary material for: Acceptability and Preferences among Men and Women for Male Involvement in Antenatal Care
Source: J Pregnancy. 2017 Jan 24;2017:4758017. doi: 10.1155/2017/4758017 (PMC5294384; doi:10.1155/2017/4758017)
Supplement: Supplementary file 1 — The supplementary material provides the two sample letters that participants were shown and read. Participants chose which overall letter and which paragraphs they preferred from each letter in order to assess patient preferences for messaging around male involvement in antenatal care. [file 4758017.f1.docx]

**
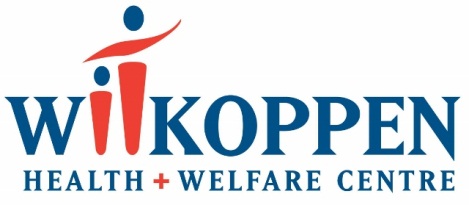
 LETTER A**

**Invitation to the Witkoppen Antenatal Clinic**

Dear___________________,

We are pleased to have met your partner today in the antenatal clinic (ANC). Congratulations on the new family member you will soon be having! To help you prepare to welcome your child, we would like to formally to invite you to the next antenatal visit with your partner.

**Why should you come for the visit?**

Your child’s health also depends on you, even before your child has been born. A good father supports his partner in attending ANC and getting tested along with her.

**What will happen during the visit?**

We will provide special counselling for fathers who are expecting so that you know how to support your partner and your new baby.

You will be offered testing for HIV and also other Sexually Transmitted Infections (STI)

You will receive counselling on how to keep yourself and your child healthy and prevent your child from getting HIV.

We look forward to seeing you soon!

Sincerely,

Name:

Title:

**
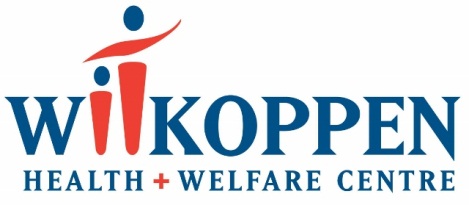
 LETTER B**

**Invitation to the Witkoppen Antenatal Clinic**

Dear___________________,

We are pleased to have met your partner today in the antenatal clinic (ANC). Congratulations on the new family member you will soon be having! To help you prepare to welcome your child, we would like to formally to invite you to the next antenatal visit with your partner.

**Why should you come for the visit?**

Research shows that when fathers attend antenatal care with their partners, both mothers and babies have better health outcomes. Your child’s health also depends on you, even before your child has been born.

**What will happen during the visit?**

You will accompany your partner and learn about your baby. We will provide special counselling for fathers who are expecting so that you know how to support your partner and your new baby.

Your health affects your ability to be a good parent. Thus, along with making sure that your partner and child are healthy, we will offer you a basic health assessment, including blood pressure reading and screening for infections that can be treated.

**Come to the clinic and give your baby the best chance at a healthy start. We look forward to seeing you soon!**

Sincerely,

Name:

Title:
